# *Paravietnura* gen. n., a new intriguing genus of Neanurini from the Caucasus (Collembola, Neanuridae, Neanurinae)

**DOI:** 10.3897/zookeys.739.22041

**Published:** 2018-02-22

**Authors:** Adrian Smolis, Nataliya Kuznetsova

**Affiliations:** 1 Institute of Environmental Biology, Department of Invertebrate Biology, Evolution and Conservation, University of Wrocław, Przybyszewskiego 65, 51-148 Wrocław, Poland; 2 Institute of Biology and Chemistry, Moscow State Pedagogical University, Moscow 129164, Russia

**Keywords:** *Paravietnura notabilis* sp. n., *Paravietnura insolita* sp. n., Russia, springtails, taxonomy

## Abstract

*Paravietnura* **gen. n.** is described and established for two new species of Neanurini from the Caucasus. The new genus is characterized by an unusual combination of features: the fusion of all lateral tubercles on the head into a single mass, the strong reduction of chaetae on the head, the fusion of cephalic tubercles Af and Oc into a transverse band, the absence of labial chaetae f, the presence of microchaetae on furca rudimentary, and the penultimate abdominal tergum with two tubercles separated along the midline. *Paravietnura* **gen. n.** strongly resembles *Vietnura* Deharveng & Bedos, 2000, a monotypic genus up to date known only from Vietnam. The main characteristics of *Paravietnura notabilis* **sp. n.** include an ogival labrum, the absence of chaetae A on the head, relatively thick and widely sheathed long macrochaetae, and minute microchaetae without chaetopores on furca rudimentary. *Paravietnura insolita* **sp. n.** differs from the previous species in chaetotaxic details, the size of furcal microchaetae, and the shape of the labrum. Short comments on the generic diversity of the tribe in the Western Palaearctic are also provided.

## Introduction

The subfamily Neanurinae, with more than 800 recognized species, is certainly one of the richest and most diversified taxa among springtails (Collembola). It is also one of the most recognizable ones within Collembola as its members possess very characteristic cuticular tubercles on the dorsal side of the body and completely lack the furca, the organ typical for most described springtails. Regarding the taxonomy of Neanurinae, all species are classified into six established tribes (Cassagnau 1989). Among them, Neanurini is the second largest, after Paleonurini, with over 170 species belonging to 23 genera (Cassagnau 1989, Hopkin 1997, Deharveng and Bedos 2000, Deharveng et al. 2007, Smolis 2007, 2011, Mayvan et al. 2015). The number of eyes, the shape of mandibles, as well as the number and arrangement of tubercles on the body are typically used to separate the genera within the tribe. In the lateral part of the head, for instance, there are four main types of tubercle arrangements. The commonest situation is with lateral tubercles L and So fused but tubercle Dl separate. In turn, a complete fusion of all lateral tubercles seems to be the rarest option, only observed in two species of Neanurini, *Vietnura caerulea* Deharveng & Bedos, 2000, and *Monobella cassagnaui* Deharveng, 1981.

The examination of rich Neanurinae materials from the north-western Caucasus has revealed two unknown species. They belong to the mentioned tribe because of a presence of blue hypodermic pigment, the last abdominal segment bilobed and well developed tubercles on the body. Interestingly, both the undescribed taxa are characterized by the complete fusion of the outlined cuticular structures on the lateral part of head. This and other features of these species support proposal of a new genus within the tribe. Its detailed and illustrated description is provided with comments on the present stage of knowledge on Neanurini diversity in the Western Palaearctic region.

## Terminology

Terminology for the description follows that of Deharveng (1983), with rationale for the definition of chaetae categories), Deharveng and Weiner (1984), Smolis and Deharveng (2006) and Smolis (2008).

### Abbreviations used

General morphology:

**Abd**. abdomen,

**Ant**. antenna,

**AOIII** sensory organ of antennal segment III,

**Cx** coxa,

**Fe** femur,

**Scx2** subcoxa 2,

**T** tibiotarsus,

**Th**. thorax,

**Tr** trochanter,

**VT** ventral tube.

Groups of chaetae on body excluding antennae:

**Ag** antegenital,

**An** chaetae of anal lobes,

**Fu** furcal,

**Ve** ventroexternal,

**Vea** ventroexternoanterior,

**Vem** ventroexternomedial,

**Vep** ventroexteroposterior,

**Vel** ventroexternolateral,

**Vec** ventroexternocentral,

**Vei** ventroexternointernal,

**Vi** ventrointernal,

**Vl** ventrolateral.

Groups of chaetae on antennae:

**ap** apical,

**ca** centroapical,

**cm** centromedial,

**cp** centroposterior,

**d** dorsal,

**vc** ventrocentral,

**ve** ventroexternal,

**vi** ventrointernal.

Tubercles:

**Af** antenno–frontal,

**Cl** clypeal,

**De** dorsoexternal,

**Di** dorsointernal,

**Dl** dorsolateral,

**L** lateral,

**Oc** ocular,

**So** subocular.

Types of chaetae:

**Ml** long macrochaeta,

**Mc** short macrochaeta,

**Mcc** very short macrochaeta,

**me** mesochaeta,

**mi** microchaeta,

**ms** ,

**S** or **s** chaeta s,

**bs** s–chaeta on Ant. IV,

**miA** microchaetae on Ant. IV,

**iv** ordinary chaetae on ventral Ant. IV,

**or** organite of Ant IV,

**brs** border s–chaeta on Ant. IV,

**i** ordinary chaeta on Ant. IV,

**mou** cylindrical s–chaetae on Ant. IV (“soies mousses”),

**x** labial papilla x,

**L**’ ordinary lateral chaeta on Abd. V,

**B4**, **B5** ordinary chaetae on tibiotarsi,

**sgv** s–chaeta on Ant. III.

## Materials and methods

The specimens were cleared in Nesbitt’s fluid, subsequently mounted on slides in Phoera liquid and studied using a Nikon Eclipse E600 phase contrast microscope. Figures were drawn with camera lucida and prepared for publication using Adobe Photoshop CS3.

### Institutions of depository of materials:

**DIBEC** Department of Invertebrate Biology, Evolution and Conservation, Institute of Environmental Biology, University of Wrocław, Poland.

**MSPU** Moscow State Pedagogical University, Institute of Biology and Chemistry, Moscow, Russia.

## Taxonomy

### Paravietnura gen. n.

Taxon classificationAnimaliaCollembolaNeanuridae

http://zoobank.org/0785483E-E218-48BB-8DD9-19BAEE8E6D0A

#### Type species.

*Paravietnura notabilis* sp. n. (here designated).

#### Diagnosis.

Blue pigment present on the body. Tubercles on body well developed, free chaetae on abdomen absent. Two pigmented eyes on each side of head. Mouth parts reduced, maxilla styliform, mandible thin and tridentate. Six tubercles on head, with Dl fused to (L + So), Af fused to Oc, and Di fused to De. Chaetotaxy of head strongly reduced, with chaetae C, D, E, Oca, Di2 and De2 absent. Labrum with four or two prelabral chaetae. Labial chaetae f absent. Tubercles (Di+De+Dl) on Abd. V separate along midline. Cryptopygy present as Abd. VI poorly visible from dorsal side. Claw untoothed.

#### Etymology.

The name “*Paravietnura*” refers to its strong similarity to *Vietnura*.

#### Remarks.

The following characters: the presence of 2+2 eyes, the fusion of all lateral tubercles into a single mass on the head, the fusion of cephalic tubercles Af and Oc into a transverse band, the absence of cephalic chaetae Di2 and De2 and the presence of strong cryptopygy place *Paravietnura* gen. n. very close to *Vietnura*, the genus established by Deharveng and Bedos (2000) for a single Vietnamese species, *V. caerulea* Deharveng & Bedos, 2000. As both these genera are distributed in separate biogeographical regions, *Paravietnura* gen. n. in the Western Palaearctic and *Vietnura* in the Oriental region, it is recognized that this similarity is probably the result of convergence. Furthermore, they differ in a few essential characters, important from the taxonomic point of view: presence/absence of cephalic chaetae Ocp (in *Paravietnura* gen. n. present, in *Vietnura* absent), presence/absence of labial chaetae f (absent in *Paravietnura* gen. n., Figs 18, 19; present in *Vietnura*, Fig. 21), the number of tubercles on Abd. V (two tubercles (Di+De+Dl) in *Paravietnura* gen. n.; three tubercles: 2 (De+Dl) and (Di+Di) in *Vietnura*), and presence/absence of microchaetae on furca rudimentary (present in *Paravietnura* gen. n.; absent in *Vietnura*).

Because of the fusion of lateral tubercles on head, *Paravietnura* gen. n. resembles *Monobella cassagnaui* Deharveng, 1981, the species belonging to the European genus *Monobella* Cassagnau, 1979. Nevertheless, this similarity seems to be definitely superficial given the fact that *M. cassagnaui* differs from *Paravietnura* gen. n. in a number of characters e.g.: fusion of tubercles (Di+De) on head along midline (separate in *Paravietnura* gen. n.), presence of chaetae Di2 and De2 on head (absent in *Paravietnura* gen. n.), fusion of tubercles Di and De on Th. II–III and Abd. I–II (separate in *Paravietnura* gen. n.), fusion of tubercles Di on Abd. III along midline (separate in *Paravietnura* gen. n.) and presence of one tubercle (2Di+2De+2Dl) on Abd. V (two tubercles (Di+De+Dl) in *Paravietnura* gen. n.).

### Paravietnura notabilis sp. n.

Taxon classificationAnimaliaCollembolaNeanuridae

http://zoobank.org/62DA9C9D-CFE3-495C-BC51-CBDE1DC22BF9

Figs 1–7 , 8 , 19 , 20 Table 1 , 2 , 3

#### Type material.

Holotype: female on slide, Russia, NW Caucasus, Adygeya, Caucasus Nature Reserve, Lagonaki Plateau (‘Kamennoye More’ ridge), 1843 m.alt., litter of rocky pine forest with birch, N44.06159° E40.02103°, 03.07.2014, leg. M. Potapov, N. Kuznetsova, A. Kremenitsa 2014, leg. (MSPU). Paratype: juvenile on slide, ibid., southern slope, 1847 m alt., mixed forest (pine, birch), coniferous litter, N44.06096° E40.02112°, 21.07.2015, leg. M. Potapov, N. Kuznetsova, A. Kremenitsa, L.Vanyavina (DIBEC).

#### Etymology.

The name reflects the notable morphology of this new generotype species.

#### Diagnosis.

Body stumpy and relatively short. Macrochaetae long, thick and widely sheathed. Buccal cone long, labrum ogival. Labrum with two prelabral chaetae. Tubercle (Af +2Oc) on head with chaetae B, Ocm and Ocp, chaetae A absent. Tubercles (Dl+L+So) on head with ten chaetae, chaetae So2 absent. Furca rudimentary with minute and difficult to detect microchaetae, without chaetopores.

#### Description.

**Figures 1–7.**
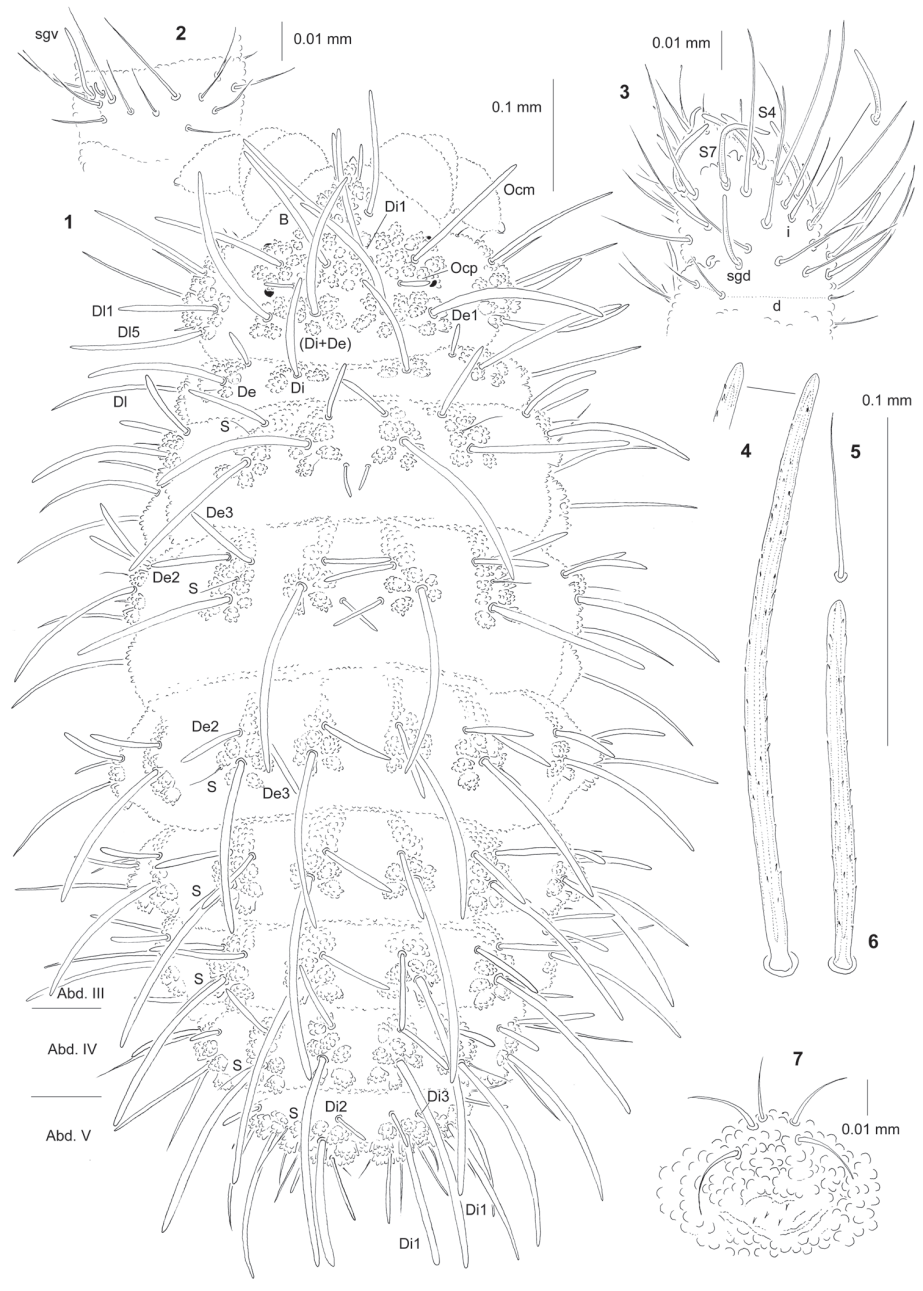
*Paravietnura notabilis* sp. n.: **1** habitus and dorsal chaetotaxy (holotype) **2** ventral chaetotaxy of Ant. III **3** dorsal chaetotaxy of Ant. III–IV **4** chaeta Di1 of Abd. IV **5** sensillum of Abd. V **6** chaeta Di1 of Abd. V **7** furca rudimentary.

*General.* Body length (without antennae): 0.55 (juvenile) to 0.85 mm (holotype). Colour of the body bluish. 2+2 black eyes of medium size (Fig. 1).

*Chaetal morphology.* Dorsal ordinary chaetae of four types: long macrochaetae (Ml), short macrochaetae (Mc), very short macrochaetae (Mcc) and mesochaetae. Long macrochaetae of large length (distinctly longer than length of segment), thick, slightly arc-like or straight, widely sheathed, strongly serrated and apically rounded (Figs 1, 4, 6). Macrochaetae Mc and Mcc morphologically similar to long macrochaetae, but much shorter. Mesochaetae similar to ventral chaetae, thin, smooth and pointed. S–chaetae of tergites thin, smooth, and very short, from three to six times shorter than nearby macrochaetae (Figs 1, 5).

*Antennae*. Dorsal chaetotaxy of Ant. III–IV as Fig. 3 and Table 2. S–chaetae of Ant. IV long and moderately thickened, S4 and S7 slightly longer than others (Fig. 3). Apical vesicle distinctly bilobed. Ventral chaetotaxy of Ant. III as Fig. 2 and Table 2, sensillum sgv notably long and s-shaped.

*Mouthparts*. Buccal cone particularly long with labral sclerifications ogival (Fig. 20). Labrum chaetotaxy: 2/2, 4. Labium with three basal, three distal, and three lateral chaetae, papillae x absent (Fig. 19). Maxilla styliform, mandible thin and tridentate.

*Dorsal chaetotaxy and tubercles*. Chaetotaxy of head as Fig. 1 and Table 1. Chaetotaxy of Th. and Abd. as Table 3. Thorax and abdomen with chaetae De2 and De3 not free (Fig. 1). On Abd. I–III, the line of chaetae De1–chaeta s perpendicular to the dorsomedian line. On Abd. IV chaetae Di1 distinctly longer than on Abd. V (Figs 1, 4, 6). On Abd. V chaetae Di3 present or absent (Fig. 1). Cryptopygy present, strongly developed.

*Ventral chaetotaxy.* On head, groups Vea, Vem and Vep with 3, 2, 4 chaetae respectively. Group Vi on head with six chaetae. On Abd. IV, furca rudimentary with minute microchaetae (Fig. 7). On Abd. IV, tubercle L with five chaetae. On Abd. V, chaetae L’ absent.

*Legs*. Chaetotaxy of legs as in Table 3. Claw without internal tooth. On tibiotarsi, chaeta M present and chaetae B4 and B5 relatively short and pointed.

**Table 1.** Cephalic chaetotaxy of *Paravietnura notabilis* sp. n., dorsal side.

Tubercle	Number of chaetae	Types of chaetae	Names of chaetae
Cl	4	Ml	F
		Mc	G
(Af+2Oc)	6	Ml	B, Ocm
		Mc	Ocp
(Di+De)	2	Ml	Di1, De1
(Dl+L+So)	10	Ml	Dl1, Dl5, L1, So1
		Mc	L4
		Mcc	Dl4
		me	So3–6

**Table 2.** Antennal chaetotaxy of *Paravietnura notabilis* sp. n.

Segment, Group	Number of chaetae	Segment, Group	Number of chaetae adult
I	7	IV	or, 8 S, i, 12 mou, 6 brs, 2 iv
II	11		
IIIve	5 sensilla AO III		
	5	ap	8 bs, 5 miA
vc	4	ca	2 bs, 3 miA
vi	4	cm	3 bs, 1 miA
d	4	cp	8 miA, 1 brs

**Table 3.** Postcephalic chaetotaxy of *Paravietnura notabilis* sp. n.

Terga					Legs				
	Di	De	Dl	L	Scx2	Cx	Tr	Fe	T
Th. I	1	2	1	-	0	3	6	13	19
Th. II	3	2+s	3+s+ms	3	2	7	6	12	19
Th. III	3	3+s	3+s	3	2	8	6	11	18
					**Sterna**				
Abd. I	2	3+s	2	3	VT: 4				
Abd. II	2	3+s	2	3	Ve: 4; chaeta Ve1 absent				
Abd. III	2	3+s	2	3	Vel:4; Fu: 5 me, 4 mi				
Abd. IV	2	2+s	3	5	Vel: 4; Vec: 2; Vei: 2; Vl: 4				
Abd. V	6–7+s				Ag: 3; Vl: 1				
Abd. VI		7			Ve: 9–10; An: 2mi				

#### Remarks.

See Remarks of *Paravietnura insolita* sp. n.

#### Ecological note.

**Figure 8.**
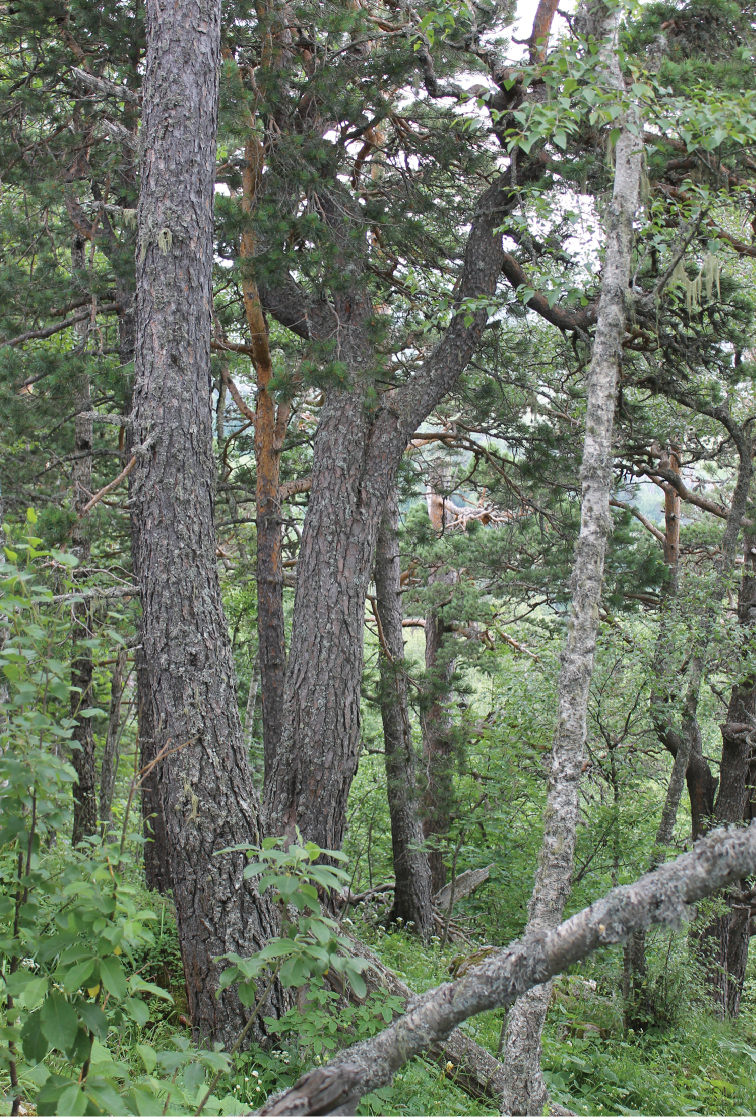
Rocky pine-birch forest in Caucasus Nature Reserve, type locality of *Paravietnura notabilis* sp. n.

The new species seems to be very local and connected with specific climatic or vegetation conditions (rocky pine-birch forest, Fig. 8) since it has been never recorded outside *locus typicus* despite many investigations conducted in different parts of the Caucasus.

### Paravietnura insolita sp. n.

Taxon classificationAnimaliaCollembolaNeanuridae

http://zoobank.org/20AB2ACF-C891-4361-9EF7-87B68DB1B127

Figs 9–18 , Table 4 , 5 , 6

#### Type material.

Holotype: male on slide, Russia, Caucasus, Northern Ossetia, North Ossetia Nature Reserve, surroundings of the village Tsey, Kalpersky ridge, southern slope, 2160 m alt., litter of rocky pine grass forest, 19.8.1977, leg. M. Rudakovsky, N. Kuznetsova (MSPU). Paratype: male on slide, ibid., green moss pine forest, in mosses, 23.9.1980, leg. I. Kuchiev (DIBEC).

#### Etymology.

Its name reflects a later discovery of another species within the genus (Latin word “*insolita*” means not lonely).

#### Diagnosis.

Body relatively short and squarish. Macrochaetae long, relatively thin and narrowly sheathed. Buccal cone rather short, labrum non ogival. Labrum with four prelabral chaetae. Tubercle (Af +2Oc) on head with chaetae A, B, Ocm and Ocp. Tubercles (Dl+L+So) on head with eleven chaetae, chaetae So2 present. Furca rudimentary with well visible microchaetae, with chaetopores.

#### Description.

**Figures 9–21.**
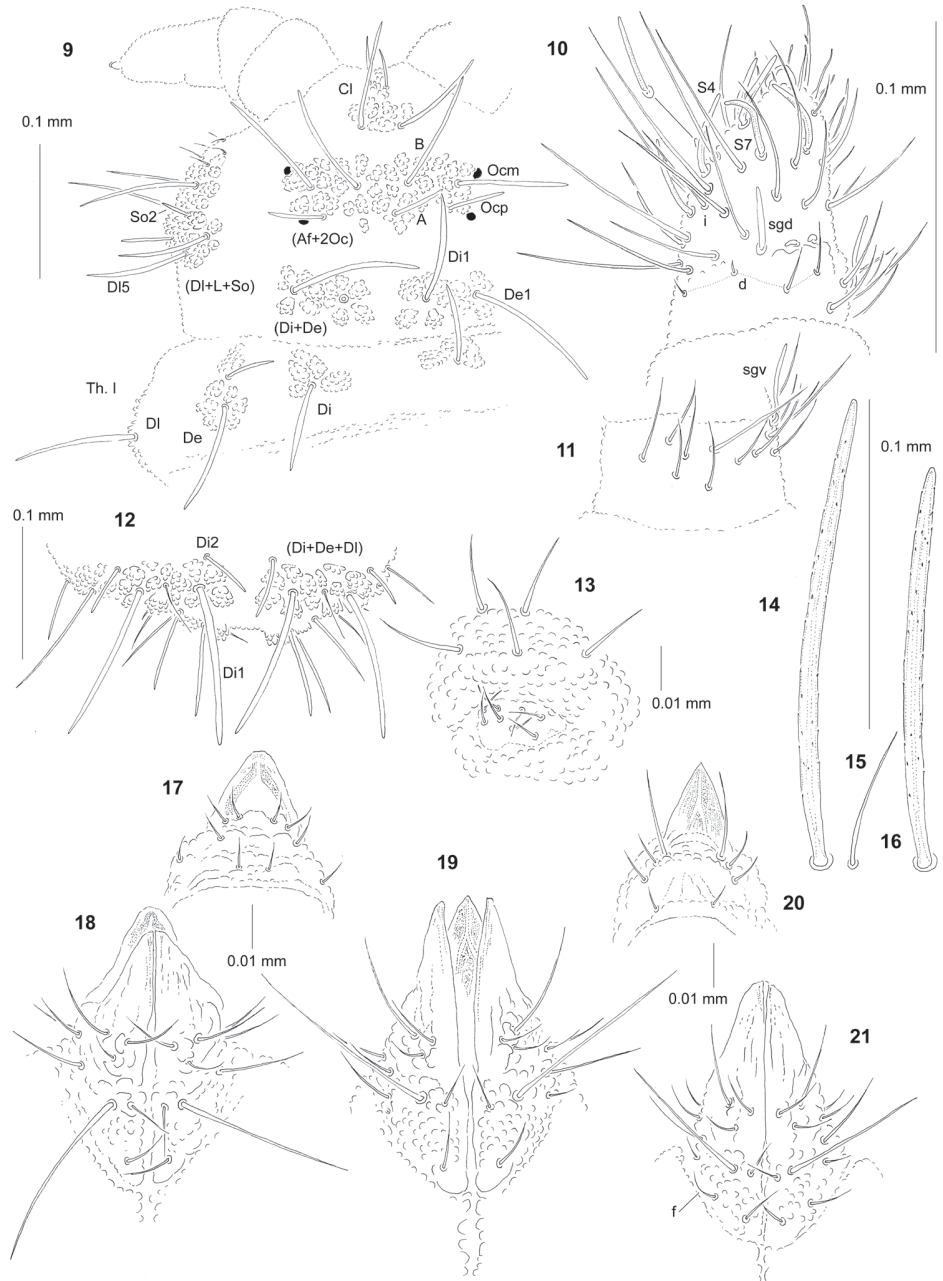
*Paravietnura insolita* sp. n.: **9** dorsal chaetotaxy of head and Th. I (holotype) **10** dorsal chaetotaxy of Ant. III–IV **11** ventral chaetotaxy of Ant. III **12** dorsal chaetotaxy of Abd. V–VI **13** furca rudimentary **14** chaeta Di1 of Abd. IV **15** chaeta Di1 of Abd. V **16** sensillum of Abd. V **17** labrum **18** labium. *Paravietnura notabilis* sp. n.: **19** labium **20** labrum. *Vietnura caerulea* Deharveng and Bedos, 2000: **21** labium.

*General.* Body length (without antennae): 1.10 (paratype) to 1.35 mm (holotype). Colour of the body bluish. 2+2 black eyes of medium size (Fig. 9).

*Chaetal morphology.* Dorsal ordinary chaetae of four types: long macrochaetae (Ml), short macrochaetae (Mc), very short macrochaetae (Mcc) and mesochaetae. Long macrochaetae of medium length (longer than length of segment), relatively thin, slightly arc-like or straight, narrowly sheathed, strongly serrated and apically rounded (Figs 9, 12, 14, 16). Macrochaetae Mc and Mcc morphologically similar to long macrochaetae, but much shorter. Mesochaetae similar to ventral chaetae, thin, smooth, and pointed. S–chaetae of tergites thin, smooth, and markedly short, three or four times shorter than nearby macrochaetae (Figs 12, 15).

*Antennae*. Dorsal chaetotaxy of Ant. III–IV as Fig. 10 and Table 5. S–chaetae of Ant. IV long and moderately thickened, S4 and S7 slightly longer than others (Fig. 10). Apical vesicle distinct bilobed. Ventral chaetotaxy of Ant. III as Fig. 11 and Table 5, sensillum sgv notably elongate and s-shaped.

*Mouthparts*. Buccal cone short with labral sclerifications non-ogival (Fig. 17). Labrum chaetotaxy: 4/2, 4. Labium with three basal, three distal and three lateral chaetae, papillae x absent (Fig. 18). Maxilla styliform, mandible thin and tridentate.

*Dorsal chaetotaxy and tubercles*. Chaetotaxy of head as Fig. 9 and Table 4. Chaetotaxy of Th. and Abd. as Table 6. Thorax and abdomen with chaetae De2 and De3 not free. On Abd. I–III, the line of chaetae De1–chaeta s perpendicular to the dorsomedian line. On Abd. IV chaetae Di1 longer than on Abd. V (Figs 14, 16). On Abd. V chaetae Di2 present and Di3 absent (Fig. 12). Cryptopygy present, well developed.

*Ventral chaetotaxy.* On head, groups Vea, Vem and Vep with 3, 2, 4 chaetae respectively. Group Vi on head with six chaetae. On Abd. IV, furca rudimentary with clearly visible microchaetae, each with chaetopore (Fig. 13). On Abd. IV, tubercle L with five chaetae. On Abd. V, chaetae L’ present.

*Legs*. Chaetotaxy of legs as in Table 6. Claw without internal tooth. On tibiotarsi, chaeta M present and chaetae B4 and B5 relatively short and pointed.

#### Remarks.

*Paravietnura insolita* sp. n. can be confused with the only other described species in the genus *Paravietnura notabilis* sp. n., which has thick and widely sheathed long macrochaetae (distinctly thinner and narrowly sheathed in *insolita*), an ogival labrum (non-ogival in *insolita*), chaetae A and So2 reduced on the head (present in *insolita*), chaetae L’ reduced on Abd. V (present in *insolita*), and the furca rudimentary with minute microchaetae without chaetopores (longer microchaetae with distinct chaetopores in *insolita*).

#### Ecological note.

Similarly to the previous species, *Paravietnura insolita* sp. n. seems to be very local and connected with specific climatic and vegetation conditions, probably with mountain xeric pine forest.

#### Variabilty.

We observed an asymmetrical absence of chaeta A in the holotype (Fig. 9).

**Table 4.** Cephalic chaetotaxy of *Paravietnura insolita* sp. n., dorsal side.

Tubercle	Number of chaetae	Types of chaetae	Names of chaetae
Cl	4	MlMc	FG
(Af+2Oc)	8	MlMc	B, OcmA, Ocp
(Di+De)	2	Ml	Di1, De1
(Dl+L+So)	11	MlMcMccme	Dl, Dl5, L1, So1L4Dl4, So2So3–6

**Table 5.** Antennal chaetotaxy of *Paravietnura insolita* sp. n.

Segment, Group	Number of chaetae	Segment, Group	Number of chaetae adult
I	7	IVap	or, 8 S, i, 12 mou, 6 brs, 2 iv
II	11		
IIIve	5 sensilla AO III		
	5		8 bs, 5 miA
vc	4	ca	2 bs, 3 miA
vi	4	cm	3 bs, 1 miA
d	4	cp	8 miA, 1 brs

**Table 6.** Postcephalic chaetotaxy of *Paravietnura insolita* sp. n.

Terga					Legs				
	Di	De	Dl	L	Scx2	Cx	Tr	Fe	T
Th. I	1	2	1	-	0	3	6	13	19
Th. II	3	2+s	3+s+ms	3	2	7	6	12	19
Th. III	3	3+s	3+s	3	2	8	6	11	18
					**Sterna**				
Abd. I	2	3+s	2	3	VT: 4				
Abd. II	2	3+s	2	3	Ve: 5; chaeta Ve1 present				
Abd. III	2	3+s	2	3	Vel: 4; Fu: 5–6 me, 8 mi				
Abd. IV	2	2+s	3	5	Vel: 4; Vec: 2; Vei: 2; Vl: 4				
Abd. V	6+s				Ag: 2; Vl: 1, L‘: 1				
Abd. VI		7			Ve: 11–12; An: 2mi				

## Discussion

As noted in the Introduction, the tribe Neanurini, containing more than 170 species in 23 genera, is the second largest within the subfamily Neanurinae. Paradoxically, in spite of such a large number of known species belonging to the tribe, knowledge about its distribution and diversity seems to be still incomplete and far from satisfactory. For example, the largest generic and species diversity of Neanurini occurs in the Western Palaearctic, where currently 18 genera and nearly 150 species are known. It should be mentioned that the tribe in this region absolutely dominates and constitutes more than 80% of all genera and species of Neanurinae. The other four Neanurinae tribes are represented only by single native species (Paranurini, Sensillanurini), single introduced species (Lobellini), and a few genera with relatively small numbers of species (Paleonurini). However, presently the picture of the generic distribution of Neanurini is highly uneven as its seven genera are known exclusively from Europe (*Albanura* Deharveng, 1982; *Cansilianura* Dallai & Fanciulli, 1983; *Catalanura* Deharveng, 1983; *Lathriopyga* Caroli, 1910; *Monobella* Cassagnau, 1979; *Neanurella* Cassagnau, 1968; *Pumilinura* Cassagnau, 1979), and the next seven taxa (*Balkanura* Cassagnau, 1979, *Cryptonura* Cassagnau, 1979; *Deutonura* Cassagnau, 1979; *Endonura* Cassagnau, 1979; *Neanura* MacGillivray, 1893; *Protanura* Börner, 1906; *Thaumanura* Börner, 1932) are present both in Europe and in areas around it, e.g., Asia Minor, the Middle East, the Caucasus, or North Africa. That means that as many as 14 genera of the tribe, of 18 known in the western part of the Palaearctic, are present in Europe. Fortunately, to better understand the distributional pattern of Neanurini, a substantial number of studies dedicated to poorly investigated areas outside the continent have been undertaken during the last three decades. They have resulted the descriptions of four unknown genera, i.e. *Caucasanura* Kuznetsova & Potapov, 1988; *Edoughnura* Deharveng, Hamra-Kroua & Bedos, 2007; *Ghirkanura* Kuznetsova & Potapov, 1988; *Persanura* Mayvan, Shayanmehr, Smolis & Skarżyński, 2015; and many new species and records of known taxa (e.g., Kuznetsova and Potapov 1988, Deharveng et al. 2007, 2015, Smolis and Kaprus’ 2009, Mayvan et al. 2015, Smolis et al. 2011, 2012, 2016a, b, c, 2017). These papers, including the work presented here, show that the diversity of Neanurini in some regions of the Western Palaearctic is still underestimated, and that this diversity can be crucial to assessing the history of this megadiverse tribe of Nenurinae.

## Supplementary Material

XML Treatment for Paravietnura

XML Treatment for Paravietnura notabilis

XML Treatment for Paravietnura insolita
